# Beyond the numbers: Critical analysis of the role of postmortem tryptase in the forensic diagnosis of anaphylaxis

**DOI:** 10.1111/1556-4029.70147

**Published:** 2025-08-06

**Authors:** Luca Tomassini, Giulia Ricchezze, Cristiana Gambelunghe, Massimo Lancia, Marco Gramaccia, Francesco De Micco, Piergiorgio Fedeli, Mariano Cingolani, Roberto Scendoni

**Affiliations:** ^1^ International School of Advanced Studies University of Camerino Camerino Italy; ^2^ Department of Law, Institute of Legal Medicine University of Macerata Macerata Italy; ^3^ Forensic Medicine, Forensic Science and Sports Medicine Section, Department of Medicine and Surgery University of Perugia Perugia Italy; ^4^ Research Unit of Bioethics and Humanities, Department of Medicine and Surgery Università Campus Bio‐Medico di Roma Rome Italy; ^5^ School of Law, Legal Medicine University of Camerino Camerino Italy

**Keywords:** aphylaxis, biomarkers, forensic pathology, immunohistochemistry, postmortem diagnosis, systematic review, tryptasean

## Abstract

The postmortem diagnosis of anaphylaxis remains a forensic challenge due to the lack of specific external signs. Tryptase, a mast cell‐derived protease, has emerged as a potential biomarker for fatal anaphylaxis. This systematic review critically examined 40 studies published between 2014 and 2024, including both biochemical and immunohistochemical analyses. Literature was identified through comprehensive searches of PubMed, Scopus, and Web of Science, following PRISMA guidelines. Biochemical studies consistently reported elevated postmortem β‐tryptase levels in anaphylaxis‐related deaths, with proposed diagnostic thresholds ranging from 30.4 to 64 μg/L. A diagnostic threshold of 53.8 μg/L demonstrated particularly strong discriminative capacity (AUC = 0.98; *p* < 0.001). Immunohistochemical analyses revealed an increased number of tryptase‐positive mast cells in lung, pharynx, and skin tissues, but standardized protocols or quantification criteria were lacking. Notably, mast cell localization around bronchioles or vascular structures was more frequent in anaphylaxis cases. Despite promising findings, substantial heterogeneity in postmortem interval, sampling site, and analytical methodology limits the generalizability of current evidence. Elevated tryptase levels were also documented in non‐anaphylactic deaths, underscoring its limited specificity. Overall, tryptase can support the postmortem diagnosis of anaphylaxis when interpreted in the context of autopsy findings, scene investigation, and clinical history. However, it should not be used as a standalone marker. Standardized protocols and combined biomarker panels are urgently needed to enhance diagnostic reliability in forensic settings.

**Highlights**

- Postmortem tryptase lacks consistent validation, limiting its use as a conclusive forensic marker.
- Cutoff levels vary widely; no consensus or multicenter validation exists.
- Immunohistochemistry lacks uniform protocols and quantification criteria.
- Diagnosing anaphylactic shock requires integrated, not standalone, investigative methods.

## 1 INTRODUCTION

Anaphylaxis is a rapid‐onset, severe systemic allergic reaction involving life‐threatening respiratory and circulatory impairment, often accompanied by skin or mucosal symptoms [1, 2]. Triggered by minimal antigen exposure in some individuals, it exemplifies a profound imbalance between the risks and benefits of immune activation [1].

Anaphylaxis involves mast cell activation via IgE antibodies, which release proinflammatory mediators [3, 4]. Among these, tryptase is a serine protease abundantly stored in mast cell granules and released in its active form during mast cell degranulation [5].

Unlike many other causes of death, anaphylaxis does not always leave macroscopically evident signs; findings may be subtle, absent, or altered by postmortem changes [6]. When present, the most common alterations involve the respiratory system, including pharyngeal or laryngeal edema, pulmonary congestion and edema, or pulmonary hyperinflation and bronchial mucus plugging—features reminiscent of a fatal asthma attack [7]. However, even these findings are non‐specific and may not be observed in all cases. In rare instances, cutaneous signs such as angioedema or erythema may be noted. Other indirect findings, such as petechial hemorrhages, cerebral edema, or organ hypoperfusion lesions, may suggest death due to shock or asphyxia, which is compatible with an anaphylactic mechanism [6, 7].

Histological support may offer additional insight, particularly when eosinophilic infiltration is documented in tissues or when mast cells exhibit histological patterns distinct from those observed in other causes of death, such as asthma [8, 9].

Since the late 20th century, tryptase has gained attention as a diagnostic tool in postmortem anaphylaxis cases [10, 11]. As a protease abundantly stored in mast cell granules, β‐tryptase is released into the circulation during mast cell degranulation and is considered a potential biochemical marker for systemic anaphylaxis [5, 12]. The detection of tryptase in postmortem serum—particularly when samples are obtained from the femoral vein using standardized protocols—has been proposed as a potentially useful tool to support the diagnosis of anaphylaxis, especially when interpreted in light of clinical and forensic findings [13, 14]. Similarly, the immunohistochemical identification of tryptase‐positive mast cells in selected tissues has been explored as a complementary approach [15, 16]. Yet factors such as postmortem interval, hemolysis, sampling site, and non‐anaphylactic triggers limit the specificity of tryptase as a standalone marker [13, 17]. Moreover, the absence of a universally accepted diagnostic cutoff further complicates its interpretation. Thus, it is essential to integrate biochemical, histological, and autopsy findings to confirm fatal anaphylaxis [14, 18].

In light of the extensive literature produced on this topic regarding the postmortem use of tryptase as a biomarker for anaphylactic death, the present review does not merely aim to verify its diagnostic utility but also seeks to critically evaluate the scientific literature published over the past 10 years, with a particular focus on studies supporting its use in forensic practice. The goal is to assess and critically examine the scientific basis for this application. This means examining available biochemical and histological data, highlighting methodological weaknesses, knowledge gaps, inconsistencies among studies, and the practical limitations of translating such evidence into autopsy diagnostics. The approach adopted is therefore critical, selective, and problem‐oriented rather than confirmatory, in line with the standards of a scientifically grounded forensic discipline.

## 2 MATERIALS AND METHODS

This systematic review followed the Preferred Reporting Items for Systematic Reviews and Meta‐Analyses (PRISMA) reporting guidelines. It has been registered with Prospero (registration number: CRD42025648861).

The PRISMA checklist is provided as Table S1.

We searched PubMed, Scopus, and Web of Science for English‐language studies on postmortem immunohistochemical diagnosis of anaphylaxis.

A broad free‐text search was used to capture varied terminology across studies on postmortem tryptase. While MeSH terms may improve specificity, a broader approach ensured no relevant studies were missed. Non‐relevant articles retrieved through this inclusive strategy were subsequently excluded during an independent screening process.

The generic free‐text search terms for PubMed were: (((((((((((((((((anaphylactic death) OR (anaphylactic reaction)) OR (anaphylactic death[MeSH Terms])) OR (anaphylactic reaction[MeSH Terms])) OR (anaphylactic‐related death)) OR (anaphylactic‐related death[MeSH Terms])) AND (postmortem diagnosis)) OR (postmortem diagnosis[MeSH Terms])) AND (forensic pathology)) OR (forensic pathologist)) OR (forensic medicine)) OR (forensic science)) OR (forsensic pathology[MeSH Terms])) OR (forensic pathologist[MeSH Terms])) OR (forensic medicine[MeSH Terms])) OR (forensic science[MeSH Terms])) AND (postmortem tryptase)) OR (postmortem tryptase[MeSH Terms]).

With regard to the Scopus and Web of Science platforms, the following search strings were used: ((((((((ALL = (anaphylactic death)) OR ALL = (anaphylactic reaction)) OR ALL = (anaphylactic‐related death)) AND ALL = (postmortem diagnosis)) AND ALL = (forensic pathology)) OR ALL = (forensic pathologist)) OR ALL = (forensic medicine)) OR ALL = (forensic science)) AND ALL = (postmortem tryptase).

The filters used for this research were set to “Humans” and “English,” with articles ranging from 2014 to 2024, including articles extracted from the references of the identified studies. Three researchers conducted independent searches across PubMed, Scopus, and Web of Science databases to identify relevant studies, while one additional researcher evaluated the selected articles to ensure they satisfied the inclusion criteria.

Extracted data included study details (authors, year, country), design, sample size, population type, histochemical techniques, studied markers, and statistical analyses. The selected articles were screened by title, abstract, methods, and keywords.

Rayyan was used to screen titles and abstracts via a semi‐automated process. It incorporates a high level of usability and can be accessed at the following website: http://rayyan.qcri.org [19, 20].

The publications finally selected for analysis had to respect the following inclusion criteria:

- Postmortem investigations;
- Forensic study;
- Study of human tissues;
- Study about immunohistochemistry;
- Biochemical studies.

Both primary studies and relevant systematic reviews and meta‐analyses were included. This decision was made to provide a comprehensive overview of the current state of knowledge and to critically integrate primary data with existing evidence syntheses. Systematic reviews and meta‐analyses were analyzed separately from primary studies to maintain distinct levels of evidence.

Additionally, letters to the editor were considered separately, taking into account their lower level of evidence, to identify the main proposals and criticisms regarding the role of tryptase in the diagnosis of anaphylactic death as expressed by the scientific community over the past decade. We deemed it appropriate to include these contributions in the interest of completeness.

Non‐inclusion and exclusion criteria were:

- Articles in languages other than English;
- Studies on tryptase conducted on living individuals;
- Studies in which anaphylactic shock is not considered.

Additional relevant articles that satisfied the inclusion and exclusion criteria were identified from the references cited in the articles retrieved through the generic free‐text search.

Literature reviews, systematic reviews, and meta‐analyses were included in the search and taken into consideration.

The study was conducted in compliance with PRISMA guidelines, employing descriptive statistics to structure and present the data. The selected articles were examined to determine the applicability of tryptase as a diagnostic marker, both in postmortem biochemical investigations and as an immunohistochemical marker, as well as to assess the extent to which this marker alone can be considered truly reliable in forensic investigations, based on the existing literature.

The data collection process encompassed both the selection of relevant studies and the extraction of pertinent data.

Three researchers independently assessed whether the articles had titles or abstracts that met the inclusion criteria, and any disagreements were resolved by achieving consensus. Two researchers extracted the relevant data, which was then reviewed by two other researchers and subsequently reconfirmed by an additional pair of investigators.

Study quality was assessed using CASP checklists, yielding an overall rigor [21]. Responses to checklist items were categorized as “Yes,” “No,” or “Can't tell,” with each item scored as 1 or 0 depending on whether the specified criteria were met. The review table (Table S2) presents a final quality score for each study, ranging from 0 to 11 (≥9 = high quality; 7–8 = moderate quality; ≤6 = low quality).

For the qualitative assessment of the selected original articles, the CASP Diagnostic Test Study Checklist was chosen, as it was deemed the most methodologically appropriate tool given the nature of the studies reviewed. Most of the included articles are forensic observational investigations conducted on autopsy populations, specifically aimed at evaluating the diagnostic validity of biochemical, histological, or immunological biomarkers (such as tryptase, IgE, degranulated mast cells, or receptor expression) in the postmortem diagnosis of anaphylactic death. Other appraisal tools, such as checklists for cross‐sectional, case–control, or cohort studies, were considered less suitable, as they do not fully capture the diagnostic focus of these studies, which are not intended to explore prevalence, risk factors, or prognosis, but rather to assess test performance (accuracy, applicability, and reproducibility).

Furthermore, a structured qualitative assessment was performed exclusively on studies explicitly classified as systematic reviews. Narrative reviews were excluded from formal methodological evaluation, as their descriptive format and absence of standardized structure rendered them unsuitable for such scoring.

Checklist items were evaluated using the categories “Yes,” “No,” or “Can't tell,” with each response assigned a score of 1 or 0 based on whether the predefined criteria were satisfied. The results are summarized in the review table (Table S2), which provides an overall quality rating for each study on a scale from 0 to 10 (≥8 = high quality; 6–7 = moderate quality; ≤5 = low quality).

The goal was not merely to collect all data on tryptase use, but to appraise each source's methodological rigor, reliability, and limitations within a forensic diagnostic framework.

## 3 RESULTS

**FIGURE 1:**
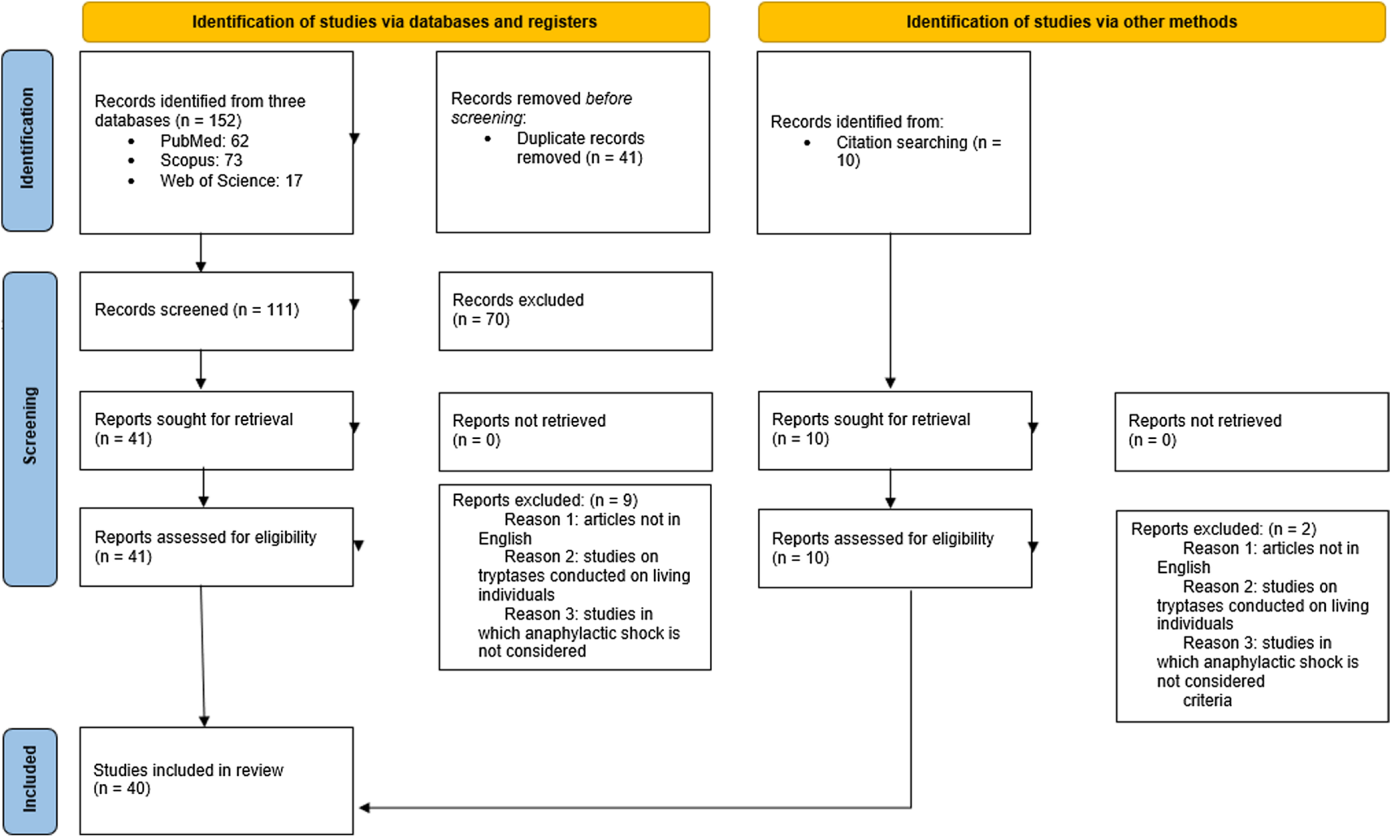
Descriptive diagram of the paper selection process.

Of 152 eligible publications, 41 duplicates were excluded. After assessing the relevance of the studies to the aims of our research, an additional 70 publications were excluded, leaving 41 full‐text articles. Upon reviewing the full‐text articles, an additional nine studies were excluded for not adhering to the inclusion criteria. The remaining 32 articles met all inclusion criteria. Furthermore, from the bibliographies of the included articles, eight more publications were selected, which also respected the established inclusion and exclusion criteria, giving a total of 40 articles. The article selection process is summarized in Figure 1.

Based on first author's affiliation, the origins of the selected studies were as follows: 14 studies from Italy, four studies from Switzerland, five studies from China, four studies from Australia, three studies from Japan, three studies from New Zealand, and one study each from Spain, Sweden, Canada, Sri Lanka, India, South Africa, and Poland.

Regarding the type of article, the selection resulted in 17 case reports, 10 original articles, three review articles, two systematic reviews, one short report, one editorial, and six letters to the editor.

### 3.1 Original articles

The original articles included one prospective case–control study [13], eight retrospective case–control designs, and one retrospective observational study.

Four studies assessed tryptase in biological fluids; two combined fluid and tissue immunohistochemistry, and four used tissue immunohistochemistry and immunofluorescence.

Regarding the cases examined, nine studies focused on anaphylactic deaths: In one study, they were compared with cardiac deaths; in another, with control cases consisting of deaths due to acute intoxication from substance abuse; and in seven studies, anaphylactic deaths were compared with various other causes of death. One study reported a retrospective series of suspected anaphylactic deaths.

In two studies, deaths were attributed to anaphylaxis caused by contrast media; in three studies, the anaphylactic reactions were traced back to drugs. One study linked anaphylactic deaths to multiple causes: Hymenoptera stings, drugs, and food. In another single study, multiple causes were again reported, specifically Hymenoptera stings, injected medications (β‐lactams), and contrast media. In two studies, the anaphylaxis trigger was unspecified.

Among the studies in which biochemical analysis was performed, five studies considered only tryptase (referring at least to the β‐isoform), while in three studies, the biochemical analysis of biological fluid(s) also included other markers, the details of which are provided in Table S3.

In four studies, tryptase was measured in blood from the femoral vein; in one study, samples were obtained from right heart chambers in addition to pericardial fluid; in another, blood was collected from the femoral vein as well as pericardial fluid, urine, and vitreous humor. One sampled only the femoral artery; another did not specify the sampling site or fluid.

Immunohistochemistry/immunofluorescence studies sampled tissues from the spleen (two); stomach, jejunum, lungs, heart, and larynx (one); throat, lungs, and intestine (one); lungs alone (one); and lungs, glottis, and skin (one). Further details for each study are provided in Table S3.

It should be noted that, among the studies employing immunohistochemistry and/or immunofluorescence, four used only anti‐tryptase antibodies; in one study, anti‐IgE antibodies were also used, while another study employed antibodies targeting FcεRIα, as well as anti‐tryptase antibodies.

Of the four biochemical studies, two did not report the postmortem interval or body storage conditions. One study sampled femoral blood 12–72 h postmortem; bodies were refrigerated within 24 h, at 4°C; in another study, blood was collected within 24 h of death “under cold chain conditions.”

Of the two IHC/IF studies, one used bodies within 24 h postmortem; the other, 0–85 days. In the former, bodies were frozen within 6 h after death, whereas in the latter, no information was provided regarding storage conditions.

With regard to the studies that employed both immunohistochemical/immunofluorescence techniques and biochemical analyses, in one study, the autopsy was performed within 48 h postmortem; in another, the sampling was carried out within 72 h; and in a third, samples were collected within 4 days. In the last one, no specific details were provided about the sampling methods. Only one of the four reported the storage temperature.

The results concerning tryptase quantification revealed the following findings.

Significant β‐tryptase elevation was found only in anaphylaxis deaths, not in cardiac controls [13]. Similar findings were reported in another study [22].

In partial contrast, postmortem biochemical analyses revealed a significant increase in serum β‐tryptase levels in six cases, suggesting an anaphylactic reaction as the cause of death—particularly following the administration of contrast media [14]. In these individuals, β‐tryptase concentrations in pericardial fluid were also elevated, exceeding both clinical reference values (11.4 ng/mL) and postmortem thresholds (45 ng/mL), with peaks reaching up to 119 ng/mL. By contrast, vitreous humor and urine levels stayed below clinical thresholds. However, elevated β‐tryptase levels in both serum and pericardial fluid were also observed in certain non‐anaphylactic deaths, such as those related to heroin overdose, diabetic ketoacidosis, myocardial infarction, and trauma, although the increases were generally less pronounced than in allergic reaction cases. By contrast, in deaths from non‐allergic causes such as hypothermia, firearm suicide, or sudden death with minimal atherosclerosis, tryptase levels were consistently low across all analyzed fluids. Furthermore, Comment et al. [14] also observed that in bodies in advanced stages of decomposition, even in the absence of allergic reactions as the cause of death, β‐tryptase concentrations in the pericardial fluid could be markedly elevated (exceeding 5000 ng/mL), suggesting that decomposition may lead to significant postmortem release of tryptase in this area.

Other research found significantly higher postmortem tryptase levels in anaphylaxis deaths than in other groups. In cardiac deaths, values varied depending on coronary syndrome status. Elevated tryptase was also seen in some cases of dissecting aneurysm, with no correlation to sex, age, or postmortem interval [23].

High tryptase levels were confirmed in anaphylaxis cases, with deaths occurring within 1–6 h postexposure. A threshold of 53.8 μg/L was proposed (AUC ROC = 0.98). Age, sex, and resuscitation did not influence values, while the effect of postmortem interval remained unclear [24].

A threshold of 64 μg/L was proposed based on 122 cases (46 confirmed), showing 95.5% specificity and 74.4% sensitivity. Tryptase levels were higher in Hymenoptera and drug‐induced reactions, and lower in food‐induced cases, although low levels did not exclude anaphylaxis [18].

Biochemical analyses revealed postmortem elevations in both serum β‐tryptase and total IgE beyond reference values, sometimes accompanied by specific IgE against insect venom, thereby supporting an anaphylactic cause of death, especially when autopsy findings excluded other causes. In the control group, β‐tryptase levels generally remained within the reference range (13.5 μg/L), with rare exceptions unrelated to anaphylaxis. Total IgE was elevated in only a few isolated cases, without concurrent increases in β‐tryptase [25].

A review of 11 cases using both biochemical and immunohistochemical testing consistently reported tryptase >40 μg/L and proposed a diagnostic algorithm [26].

In tissue‐based studies, degranulated mast cells were found in spleen samples [14], and strong tryptase staining was observed in the stomach, jejunum, lungs, larynx, and heart, while controls showed minimal staining [22]. Other studies demonstrated colocalization of FcεRIα and tryptase with statistically significant differences [15], as well as the presence of numerous mast cells and IgE‐positive cells in allergic individuals, especially in the pharynx [16]. Mast cell degranulation was prominent in anaphylactic deaths and correlated with high concentrations of tissue‐bound IgE. Increased eosinophils, mast cells, and degranulated mast cells were also observed in anaphylaxis cases, without notable differences based on allergen type (e.g., drugs, insect stings, contrast agents) [25]. Although less sensitive than immunohistochemistry, Pagoda Red staining proved useful as a preliminary diagnostic tool. The use of anti‐tryptase antibodies in lung, glottis, and injection‐site tissues showed strong positivity in anaphylaxis cases (*p* < 0.05), confirmed by optical and confocal microscopy [26].

### 3.2 Qualitative analysis of original articles

As described in the Section 2, the qualitative evaluation of the original articles was carried out using the CASP Diagnostic Test Study Checklist, chosen for its methodological alignment with the specific diagnostic focus of the studies included. All original studies in this review were assessed within a unified diagnostic framework, as they shared a common objective: to evaluate the diagnostic accuracy of postmortem biomarkers in the context of anaphylaxis‐related deaths.

Each study was evaluated using 11 checklist items, with answers categorized as “*Yes*,” “*No*,” or “*Can't tell*.” For scoring purposes, a point was awarded for each “Yes” response, while “No” and “Can't tell” received zero points. This process yielded a final score between 0 and 11, allowing for an overall methodological quality assessment. Studies scoring nine or more points were of high quality, those with seven to eight points were rated as moderate quality, and those with six or fewer points were classified as low quality.

The criteria for evaluating studies were applied to 10 studies. None of them were classified as “High quality,” seven were rated as “Moderate quality,” and three were categorized as “Low quality.”

### 3.3 Case reports

In total, 20 cases were reviewed: 15 out of 17 articles described a single case report [27], one article presented two cases [28], and another article reported a total of three cases [29].

Anaphylaxis was suspected to be food‐related in five cases [30], attributed to arthropod stings in six [31, 32], and linked to drug exposure in another six [33]. Additionally, one case involved suspected anaphylaxis following a sting from a crown‐of‐thorns starfish (*Acanthaster planci*); in another, the reaction was thought to have resulted from the transfer of peanut allergens during oral contact between two adolescent males [34]; and in the last case, intravenous administration of an aqueous cannabis extract was identified as the trigger.

Regarding the type of investigation, 13 reports focused exclusively on tryptase quantification in biological fluids (mostly serum and blood, with a single case involving vitreous humor). The remaining reports included tryptase quantification in biological fluids and also examined its use as an immunohistochemical marker in tissue samples.

In 16 reports, the primary suspected diagnosis was anaphylaxis, whereas in the remaining four cases, the diagnostic work‐up mentioned eosinophilic coronary periarteritis [35], underlying indolent mastocytosis, Kounis syndrome [36], and α‐gal syndrome [37].

Concerning the timing of sample collection, six reports did not specify the time interval between death and the collection of fluids or tissues, nor the postmortem conditions under which the body was kept. In the remaining cases, sampling was performed at various time intervals after death: in one case, 45 min post‐cardiac arrest; in one case, 5 h postmortem; one at 6 h; one at 15 h; one at 24 h; one at 28 h; one at 2 days; one at 72 h; one at 42 h; one at 60 h; and two at approximately 96 h (one of which referred to “4 days”) [38]. In one report, sampling was explicitly stated to have occurred 96 h after death. In two cases, two separate postmortem samplings were performed, one at 3 days and the other at 6 days after death in the first, and one at 2 days and the other at 3 days after death in the second.

In 16 reports, postmortem quantification of immunoglobulin E (IgE) was conducted as an additional analysis alongside tryptase determination. In the remaining four reports, IgE was not mentioned.

Regarding immunohistochemistry, among the seven reports examined, four employed anti‐CD117 immunostaining, one used c‐kit, and one used anti‐CD3 and anti‐CD8 antibodies, the latter to support a diagnosis of celiac disease.

Still in the context of immunohistochemistry, anti‐tryptase antibodies were used to identify mast cells in various tissues: In two reports, pulmonary tissue was examined; in one, splenic tissue; in another, cardiac tissue; and in one case, both skin and lung tissues were analyzed. In two additional studies, immunohistochemical analysis involved the glottis, lungs, and myocardium in one case, and the larynx, lung, heart, and spleen in the other.

With regard to the results of tryptase quantification conducted on serum/blood samples, the reports in which a single sampling was performed (a total of 18 cases) consistently showed elevated tryptase concentrations compared with the reference range, except for one case [39]. In both instances where serial sampling was conducted, a decrease in blood tryptase levels was observed—over approximately 72 h (from day 3 to day 6) in one case [40], and approximately 24 h (from day 2 to day 3 postmortem) in the other [41]—although elevated levels were still present in both cases.

From an immunohistochemical standpoint, based on the studies that employed this technique, all case reports revealed the presence of tryptase‐positive cells in all examined tissues, consistent with mast cells. In a single case, a comparative count was performed at the level of the pulmonary parenchyma between the mast cells of the case in question and those of a control group composed of individuals who died from traumatic causes (although no specific details regarding the latter were provided) [42]. Further details for each study are provided in Table S4.

### 3.4 Reviews

Overall, five literature reviews met the inclusion criteria. Of these, three were narrative reviews, one was a systematic review, and one was a systematic review with meta‐analysis. The individual studies were published between 2016 and 2023: one in 2016, one in 2018, one in 2020, one in 2021, and one in 2023. Among these, three articles focused on the diagnostic investigation of anaphylaxis‐related deaths, considering various markers; the remaining two articles, including the meta‐analysis and the systematic review, specifically addressed the role of tryptase in anaphylactic fatalities.

With regard to the investigated methodologies, three reviews focused on biochemical analyses, one addressed exclusively immunohistochemical techniques, and a single review considered both biochemical and immunohistochemical approaches.

Concerning the findings from reviews conducted using systematic methods, the two systematic reviews analyzed demonstrate a degree of consensus regarding the diagnostic utility of postmortem serum tryptase and immunohistochemistry in identifying deaths due to anaphylactic shock. However, they also highlight significant methodological and interpretative limitations. One quantitative meta‐analysis confirmed a significant difference in tryptase levels between anaphylactic and non‐anaphylactic deaths, proposing a diagnostic cutoff of 30.4 μg/L [43]. Nonetheless, the high heterogeneity across studies (*I* ^2^ up to 100%) raises concerns about the reliability of the pooled estimate, making it problematic to generalize the results—particularly in forensic contexts involving decomposed samples or variable postmortem intervals [43].

The most recent systematic review [44] focused on the use of immunohistochemistry as a complementary and often decisive tool. The analysis of 162 cases revealed consistent tryptase positivity but significantly lower sensitivity for other markers such as CD117 and chymase. These findings reinforce the central role of tryptase, while also pointing to the need for additional tissue biomarkers. However, this study is also affected by heterogeneity in terms of differences in case selection and in the quality of primary sources, which are largely based on case reports or uncontrolled retrospective studies [44].

Regarding the qualitative appraisal, it should be noted that this was conducted exclusively for the systematic reviews (with or without meta‐analysis). Overall, one review was rated as moderate quality, while the other was assessed as low quality.

### 3.5 Letters

Six letters to the editor were retrieved and reviewed to critically assess recurring concerns and remarks raised in the forensic literature regarding the use of tryptase as a postmortem biomarker for anaphylactic death. Of these, one letter was a commentary in response to a literature review [45], one was a comment on a case report [17], two were responses to the same original article [24, 46, 47], one was a rejoinder to a previously mentioned letter, and one letter did not provide observations related to any specific article [48].

One letter [48] raised concern regarding the interpretive limitations of β‐tryptase postmortem values due to the wide variability of concentrations reported in both anaphylactic and non‐anaphylactic deaths. The authors stressed that β‐tryptase should be measured and interpreted in isolation rather than in combination with α‐isoforms and argued that cutoff values alone are insufficient for diagnosis without proper contextual analysis.

Similarly, another letter [46] underscored the necessity of measuring β‐tryptase exclusively, disputing claims that specific assays were not commercially available. It emphasized that β‐tryptase is more stable and diagnostically relevant than α‐tryptase due to its delayed diffusion and slower degradation postmortem, and advocated for validated, quality‐controlled protocols.

A broader perspective was offered in another letter [45], which, based on a systematic literature search, noted that many studies failed to clarify whether total or isoform‐specific tryptase was measured, and often omitted preanalytical variables such as sampling site, postmortem interval, and storage conditions. The authors proposed a standardized protocol for β‐tryptase sampling and measurement, while acknowledging that resuscitation and cause of death may influence postmortem values.

Another comment [17] reported a progressive decline in β‐tryptase levels over time in fatal anaphylaxis cases, consistent with expected biochemical kinetics rather than decomposition, thus supporting early sampling and serial measurements.

Statistical and methodological issues were raised in one letter [47], which questioned the validity of using a *p*‐value threshold of 0.1 in multivariate analysis, the choice of control groups, and the unbalanced distribution of covariates such as sex and CPR status. In reply, the authors of the original article [49] explained that their regression approach was chosen for methodological simplicity and robustness. They clarified that their goal was not to define a universal cutoff, but rather to explore contributors to tryptase variability, reaffirming the importance of interpreting tryptase levels within a broader investigative context.

## 4 DISCUSSION

Over the past decade, literature on postmortem tryptase has been abundant but methodologically inconsistent. Overall, numerous studies were identified, including original articles, case reports, reviews, and letters to the editor. However, the methodological characteristics of these studies are highly variable, primarily due to the coexistence of biochemical and immunohistochemical investigations, which sometimes overlap (even within the same study).

Methodological heterogeneity spans study design, case selection, sampling, analysis, and population types (suspected anaphylaxis, negative controls, mixed cases). This variability limits meaningful comparison and synthesis of findings.

Regarding original studies, the biochemical investigations presented in the selected articles depict a heterogeneous and complex landscape, with promising preliminary findings but also significant methodological limitations that affect the robustness of the conclusions.

Overall, the data suggest that postmortem tryptase measurement, particularly its β‐fraction, represents a potentially valuable tool in supporting the diagnosis of anaphylactic death. Studies such as those by Palmiere et al. and Xiao et al. have documented a statistically significant increase in serum tryptase levels in deaths attributed to anaphylaxis compared to those due to cardiac or mixed causes, highlighting a potentially exploitable biochemical differential [13, 23].

Similarly, the study by Tse et al. [24] proposed an operational cutoff value (53.8 μg/L) with a high discriminative capacity (AUC 0.98), further supporting the hypothesis that elevated postmortem tryptase levels may indeed indicate an acute anaphylactic event.

Closer analysis reveals several key issues. Chief among these is the extreme variability of the proposed cutoff values, ranging from approximately 30 μg/L to over 60 μg/L [18], without clear consensus or external multicenter validation.

This heterogeneity stems from differences in design, inclusion criteria, and sampling techniques. For example, Comment et al. [14] have shown that pericardial fluid can exhibit markedly elevated tryptase levels even in the absence of anaphylaxis, particularly under conditions of advanced decomposition, raising concerns about the reliability of this marker in tissues or fluids other than femoral blood.

Similar critical issues arise concerning the postmortem interval (PMI). In several studies, the time window between death and blood sampling was either wide or not adequately controlled—sometimes extending beyond 72 h, as reported in the cases presented in previous studies [40, 41].

Although a slow decline in tryptase levels after death, consistent with postmortem enzymatic degradation, has been observed, the rate and extent of this decrease remain poorly predictable and unstandardized. This adds a confounding variable, especially when sampling is delayed or preservation is poor.

Another key issue is the limited specificity of tryptase. It has been demonstrated that elevated tryptase levels can also be observed in non‐anaphylactic deaths—such as acute cardiac ischemia, severe trauma, and substance abuse‐related fatalities—as reported in previous studies [14, 23].

Elevated tryptase alone is not diagnostic of anaphylaxis and requires a clinical‐autopsy context. In this regard, the risk of false positives appears substantial, particularly in the absence of compatible histopathological or anamnestic findings.

At the analytical level, it should also be noted that several studies do not specify whether the biochemical analysis focuses exclusively on the β‐tryptase fraction, which is more indicative of acute mast cell degranulation, or on total tryptase (α + β), whose postmortem interpretation remains more controversial [49, 50]. In some studies, the search for tryptase is not restricted to the β‐tryptase isoform, which is considered to be the true marker of systemic anaphylaxis [7].

Most studies do not consistently pair tryptase with other markers (e.g., histamine, carboxypeptidase A, IgE), limiting diagnostic value. Some exceptions, such as the studies by Bonetti et al. and Esposito et al., which combine tryptase analysis with that of total and specific IgE, suggest that a combined approach could enhance diagnostic sensitivity; however, these data remain limited and require confirmation [25, 26].

In conclusion, the available biochemical literature (composed of original articles) suggests that postmortem tryptase measurement, particularly under standardized conditions and complemented by other clinical‐forensic findings, may well support the diagnosis of fatal anaphylaxis. Yet inconsistent protocols, threshold variability, PMI uncertainty, and limited specificity warrant cautious interpretation. Although promising, the available evidence does not yet allow for the sole biochemical value of tryptase to be considered decisive or sufficient in current forensic practice.

On the contrary, a critical evaluation of the immunohistochemical investigations presented in the original articles reviewed reveals a promising yet methodologically fragile and fragmented landscape. Overall, tissue tryptase immunohistochemistry may aid postmortem anaphylaxis diagnosis. Several studies have documented an increased number of tryptase‐positive mast cells in pulmonary, laryngeal, cardiac, and cutaneous tissues from individuals whose death was attributed to anaphylaxis compared with non‐anaphylactic controls. Patterns like peribronchial or mucosal infiltration may suggest systemic allergic reactions [14, 15, 22, 26].

However, a more rigorous examination of the studies reveals numerous critical issues. A key issue is poor methodological standardization: Sampled tissues vary, and site selection lacks a consistent rationale. This adds uncertainty, as mast cell density varies naturally by region [51, 52, 53].

An additional limitation concerns the technical heterogeneity of the immunohistochemical methods employed. Despite the consistent use of anti‐tryptase antibodies, studies lacked uniformity in staining, protocols, and quantification. Esposito et al. [26] are among the few to have proposed quantitative or semi‐quantitative analyses (e.g., mast cell counts per high‐power field, confocal evaluations). Most observations remain purely descriptive and qualitative, limiting the reproducibility and objectivity of the findings.

A major obstacle is the lack of standardized scoring criteria for interpreting tryptase immunoreactivity. The studies reviewed rarely specify numerical thresholds or mast cell densities considered indicative of anaphylaxis versus normal variability or other pathological conditions. This limits cross‐study comparability and keeps IHC suggestive rather than definitive.

Equally relevant is the issue of the specificity of tryptase expression. Tryptase‐positive mast cells also appear in non‐anaphylactic states like infection, inflammation, or CPR [54, 55, 56, 57, 58].

Some observations deserve particular attention. The study by Feng et al. [15], for example, proposed the combined use of immunofluorescent markers for FcεRIα and tryptase in an attempt to improve specificity in identifying activated mast cells. Although promising, this approach requires broader validation before it can be considered standard practice. Similarly, the findings reported by Wang et al. [16] regarding increased numbers of IgE‐positive mast cells in the pharyngeal region offer interesting insights but remain exploratory and based on small sample sizes.

Another critical point concerns the management of postmortem timing and tissue preservation methods. Tissue degradation can affect antigenic integrity and the quality of immunohistochemical staining. PMIs ranged from hours to 85 days, often without documented preservation conditions [16]. This introduces a potentially significant bias and makes it difficult to determine to what extent the observed findings reflect true vital phenomena rather than postmortem artifacts.

In summary, the use of tryptase immunohistochemistry in autopsy tissues constitutes a potentially useful technique in supporting the diagnosis of anaphylaxis, but at present, it still exhibits weaknesses.

Methodological variability (including the perspective of microscopic tissue examination), the absence of defined quantitative criteria, the lack of systematic controls, and the risk of overlap with non‐allergic inflammatory conditions severely limit the possibility of using immunohistochemistry as a standalone, reliable diagnostic tool.

Thus, tryptase positivity in IHC should be viewed as supportive—not standalone—evidence. Real forensic applicability requires standardized protocols, multicenter validation studies, the definition of clear quantitative scoring systems, and integration with clinical‐anamnestic, biochemical, and macroscopic data.

Tryptase and/or anaphylactic death were examined in five of the contributions selected from the relevant literature. Among these, several are narrative which, by their nature, while offering valuable theoretical insights and broad overviews, do not follow a systematic protocol for the selection, assessment, and synthesis of evidence. As such, although these narrative reviews are of significant interest, they cannot be regarded as sources of high‐level evidence [7, 8, 59].

By contrast, systematic reviews warrant a more detailed evaluation.

In this sense, the systematic review presented in a 2018 study represents the most robust contribution currently available from a methodological standpoint. Following PRISMA guidelines, the authors conducted a search across four major databases, ultimately selecting nine retrospective studies that allowed for a meta‐analysis comparing postmortem tryptase levels between anaphylactic and non‐anaphylactic deaths. The findings confirmed a significant difference, proposing a threshold value of approximately 30.4 μg/L. However, the overall methodological quality of the included studies (assessed via the Newcastle‐Ottawa scale) was moderate, and several critical issues remain: the absence of prospective studies, considerable heterogeneity in the diagnostic criteria for anaphylaxis, and a lack of standardization in sampling and preservation methods.

In this respect, another systematic review [43] offers important insights. It also highlights several limitations—notably the heterogeneity of the studies, the absence of standardized protocols, and the influence of confounding variables—which collectively and substantially restrict the real‐world applicability of tryptase as a sole reliable diagnostic marker in forensic settings, confirming the results of our work.

On the contrary, in light of the critical considerations outlined in this section regarding immunohistochemistry, we cannot fully endorse the conclusions reached in a recent study [44]. Although their systematic review offers valuable insights, the available evidence appears, in our view, insufficient for immunohistochemistry to qualify as a “fundamental tool” for anaphylaxis. Rather, it suggests that further well‐designed studies are necessary to substantiate this—an implicit need that even the authors themselves acknowledge.

Finally, the material derived from case reports and letters to the editor constitutes a valuable, albeit inherently anecdotal, testimony to the current state of forensic debate regarding the diagnosis of anaphylactic death. The case reports reviewed present a heterogeneous array of clinical and autopsy experiences, in which postmortem tryptase determination—sometimes accompanied by immunohistochemical or specific IgE analyses—proved useful within contexts of strong circumstantial and anamnestic suspicion. Nevertheless, the systematic absence of adequate control groups, variability in sampling quality and timing, and potential errors of inference highlight how the literature based on isolated cases, while driven by the desire to enhance diagnostic practice, risks generating more hypotheses than certainties. Letters to the editor, on the contrary, reveal a vibrant critical discourse: Multiple authoritative voices urge the scientific community to abandon simplistic approaches and promote rigorously standardized protocols for β‐tryptase measurement, emphasizing the need to control preanalytical variables and consistently interpret biochemical findings within a broader investigative framework.

Among the case reports, a few examples stand out for their methodological interest: Cases involving serial postmortem sampling demonstrated a gradual decline of tryptase levels over time, strengthening the interpretation of antemortem mast cell degranulation [40, 41]; comparative immunohistochemical analyses attempted to objectify mast cell density against control populations; and unusual anaphylactic triggers, such as intravenous cannabis extracts or allergen transfer through oral contact, expanded the forensic horizon beyond conventional scenarios [60]. These reports, although isolated, highlight the potential of a more dynamic, integrated, and critically cautious approach to the forensic investigation of suspected anaphylaxis.

Ultimately, both case reports and letters underscore the necessity for forensic science to stay up‐to‐date on technological advancements and for forensic scientists to remain intellectually aware of the field's epistemological limitations.

## 5 CONCLUSION

This systematic review critically evaluated the current scientific literature on the use of postmortem tryptase in both biochemical and immunohistochemical analyses to diagnose anaphylactic death. Although elevated β‐tryptase levels and positive tissue immunoreactivity may serve as useful indicators, their value as standalone diagnostic evidence remains limited. This is primarily due to the lack of standardized protocols, methodological heterogeneity, and uncertain specificity. Therefore, tryptase should currently be regarded as an ancillary tool, rather than definitive proof, particularly in forensic settings subject to judicial scrutiny.

Nevertheless, when applied with due consideration of these limitations, tryptase analysis can complement a broader, structured diagnostic process. In such contexts, and based on the reviewed literature, several practical considerations are necessary to maximize the interpretive utility of the findings.

Tryptase determination should be reserved for cases with strong circumstantial or anamnestic indicators of anaphylaxis, such as exposure to known allergens (e.g., insect stings, drugs, food), sudden collapse, or autopsy signs like laryngeal edema. It is not recommended for routine use in the absence of such elements.

The femoral vein is the preferred blood sampling site, being less prone to postmortem redistribution. Pericardial fluid may be considered in select circumstances, but it is more susceptible to false positives, particularly in decomposed bodies.

Timely sampling is essential and should occur as early as possible, ideally within 24–48 h postmortem, with the body maintained at 4°C. Delays or improper storage can degrade analyte integrity, reducing the reliability of results.

Proposed cutoff values for β‐tryptase vary considerably, from 30 to 64 μg/L. Given this variability, a cautious approach is recommended. A provisional range of 40–50 μg/L may be used, though lower levels—especially in food‐related cases—do not exclude anaphylaxis.

Histologically, the presence of tryptase‐positive mast cells in the lungs, heart, pharynx, or skin may support the hypothesis of anaphylactic death, particularly when arranged in perivascular or submucosal patterns. However, inconsistencies in tissue selection and staining protocols across studies highlight the need for standardization. Quantitative or semi‐quantitative assessment methods would enhance the reliability and reproducibility of immunohistochemical evaluations.

Crucially, both biochemical and histological findings must be interpreted within a comprehensive framework that includes case history, scene investigation, autopsy results, and (where possible) additional biomarkers such as specific IgE, histamine, or carboxypeptidase A. Interpretation in isolation risks diagnostic error.

In some cases, the combined use of multiple biomarkers or serial postmortem samples has demonstrated greater diagnostic precision. A postmortem decline in tryptase levels may indicate antemortem mast cell degranulation, adding evidentiary value when contextualized appropriately.

In conclusion, postmortem tryptase analysis is a valuable supportive tool in the forensic investigation of anaphylaxis. However, its application should be confined to integrated diagnostic strategies, where contextual and autoptic data are prioritized. Until standardized methodologies and validated thresholds are available, tryptase should not be relied upon as conclusive evidence.

Future research should focus on prospective multicenter studies to establish validated cutoff values, standardize immunohistochemical techniques, and develop integrated diagnostic algorithms. The implementation of multiparametric biomarker panels represents a crucial step toward improving diagnostic accuracy in suspected anaphylactic deaths.

## CONFLICT OF INTEREST STATEMENT

The authors have no conflicts of interest to declare.

## Supporting information


Tables S1–S4.

## Data Availability

Data sharing not applicable to this article as no datasets were generated or analysed during the current study.
