# Supplementary material for: Beyond the numbers: Critical analysis of the role of postmortem tryptase in the forensic diagnosis of anaphylaxis
Source: J Forensic Sci. 2025 Aug 6;70(6):2117–28. doi: 10.1111/1556-4029.70147 (PMC12584128; doi:10.1111/1556-4029.70147)
Supplement: Supplementary file 1 — Tables S1–S4. [file JFO-70-2117-s001.zip › jfo70147-sup-0002-TableS2-S4@JOFS 25-371_TABLE S2-S4 .docx]

TABLE S2 Summary presenting a final quality score for each study.

| **CASP Checklist For diagnostic test studies** | | | | | | | | | | | | | |
| --- | --- | --- | --- | --- | --- | --- | --- | --- | --- | --- | --- | --- | --- |
|  | **Section A: Are the results of the study valid?** | | | | | | **Section B: What are the results?** | | **Section C: Will the results help locally?** | | | |  |
|  | **Did the study address a clearly formulated research question?** | **Was there a comparison with an appropriate reference standard?** | **Did all patients get the diagnostic test and reference standard?** | **Could the results of the test have been influenced by the results of the reference standard?** | **Is the disease status of the tested population clearly described?** | **Were the methods for performing the test described in sufficient detail?** | **What are the results** | **How sure are we about the results? Consequences and cost of alternatives performed?** | **Can the results be applied to your patients/the population of interest?** | **Can the test be applied to your patient or population of interest?** | **Were all outcomes important to the individual or population considered?** | **What would be the impact of using this test on your patients/population?** | **Score** |
| **C. Palmiere et al. (2014)** | yes | yes | yes | can’t tell | yes | yes | no | can’t tell | yes | yes | no | n/a | 7 |
| **Lionel Comment (2014)** | yes | yes | yes | can’t tell | yes | yes | no | can’t tell | yes | can’t tell | no | n/a | 6 |
| **X.J. Guo et al. (2015)** | yes | yes | yes | can’t tell | yes | yes | yes | yes | yes | can’t tell | no | n/a | 8 |
| **Ning Xiao (2017)** | yes | yes | yes | can’t tell | yes | yes | yes | yes | yes | can’t tell | no | n/a | 8 |
| **R.Tse et al. (2017)** | yes | yes | yes | can’t tell | yes | yes | yes | yes | yes | can’t tell | no | n/a | 8 |
| **Miguel A. Tejedor-Alonso et al. (2020)** | yes | yes | no | can’t tell | yes | yes | yes | yes | yes | can’t tell | no | n/a | 7 |
| **Feng et al. 2021** | yes | yes | yes | no | yes | yes | no | yes | yes | can’t tell | no | n/a | 7 |
| **Bonetti et al. (2015)** | yes | yes | yes | can’t tell | yes | yes | no | yes | yes | can’t tell | no | n/a | 7 |
| **Esposito et al. (2021)** | yes | can’t tell | yes | can’t tell | yes | yes | no | can’t tell | no | can’t tell | no | n/a | 4 |
| **Wang et al. (2020)** | yes | yes | yes | can’t tell | yes | yes | no | yes | no | no | no | n/a | 6 |

| **Systematic reviews with meta-analysis of observational studies** | | | | | | | | | | | |
| --- | --- | --- | --- | --- | --- | --- | --- | --- | --- | --- | --- |
|  | **Section A: Is the basic study design valid for a systematic review?** | | **Section B: Is the systematic review methodologically sound?** | | | **Section C: Are the results of the systematic review trustworthy?** | | | **Section D: Are the results of the systematic review relevant locally?** | **Section E: Will the implementation of the results represent greater value for your service users or population?** |  |
|  | **Did the systematic review address a clearly formulated research question?** | **Did the researchers search for appropriate study designs to answer the research question?** | **Were all relevant primary research studies likely to have been included in the systematic review?** | **Did the researchers assess the validity or methodological rigour of the primary research studies included in the systematic review?** | **Did the researchers extract, and present information on the individual primary research studies appropriately and transparently?** | **Did the researchers analyse the results of the individual primary research studies appropriately?** | **Did the researchers report any limitations of the systematic review and, if so, do the limitations discussed cover all the issues in your critical appraisal?** | **Would the benefits of acting upon the results outweigh any potential disadvantages, harms and/or additional demand for resources associated with acting on the results?** | **Can the results of the systematic review be applied to your local population/in your local setting or context?** | **If actioned, would the findings from the systematic review represent greater or additional value for the individuals or populations for whom you are responsible?** | **Score** |
| **Sun et al. (2008)** | yes | yes | yes | yes | yes | yes | yes | can’t tell | can’t tell | can’t tell | 7 |

| **Systematic Review Checklist** | | | | | | | | | | | |
| --- | --- | --- | --- | --- | --- | --- | --- | --- | --- | --- | --- |
|  | **Section A: Are the results of the review valid?** | | | | | **Section B: What are the results?** | | **Section C: Will the results help locally?** | | |  |
|  | **Did the review address a clearly focused question?** | **Did the authors look for the right type of papers?** | **Do you think all the important, relevant studies were included?** | **Did the review’s authors do enough to assess quality of the included studies?** | **If the results of the review have been combined, was it reasonable to do so?** | **What are the overall results of the review?** | **How precise are the results?** | **Can the results be applied to the local population?** | **Were all important outcomes considered?** | **Are the benefits worth the harms and costs?** | **Score** |
| **Del Duca et al. (2023)** | yes | no | yes | no | yes | can’t tell | can’t tell | can’t tell | no | can’t tell | 3 |

TABLE S3 Synthesis table of the studies analyzed.

| **Authors and country** | **Number of cases/controls** | **Type of population** | **Inclusion/exclusion criteria** | **Autopsy** | **Autopsy findings** | **Histochemical staining used** | **Type of matrix studied** | **Histochemical results** | **Tryptase research** | **Tryptase values (results)** | **Reference cutoff** | **Other markers** | **Immunohistochemistry** | **Immunohistochemistry results** | **Immunofluorescence** | **Statistical analysis** |
| --- | --- | --- | --- | --- | --- | --- | --- | --- | --- | --- | --- | --- | --- | --- | --- | --- |
| Palmiere et al. (2014)  Switzerland (13) | 96 subjects | Group A: 30 cases of individuals who appeared healthy and died suddenly. Postmortem examinations revealed only minimal coronary atherosclerosis. The cause of death was attributed to cardiac arrhythmia or cardiac arrest, with no macroscopic or microscopic abnormalities identified apart from minimal coronary artery atherosclerosis. Group B: 30 cases involving individuals with significant coronary artery atherosclerosis, including extensive calcifications and severe luminal narrowing. Myocardial fibrosis was also observed during postmortem examination. There was no evidence of acute coronary thrombosis or myocardial infarction. The cause of death was attributed to cardiac arrhythmia or cardiac arrest occurring in the context of advanced coronary artery atherosclerosis and myocardial fibrosis. Group C: 30 cases involving individuals with coronary artery atherosclerosis, acute coronary thrombosis, and acute myocardial infarction identified during postmortem examination. The cause of death was determined to be myocardial infarction resulting from acute coronary thrombosis. Group D: 6 cases involving fatal hypersensitivity reactions following exposure to contrast media, all of which were subject to medicolegal investigation. The cause of death was identified as anaphylactic shock. | Postmortem interval not exceeding 48 hours. Availability of femoral blood, postmortem serum from femoral blood, and vitreous humor.  No signs of advanced decomposition. | Autopsies were performed within 24 hours of the discovery of the body | Not mentioned | Hematoxylin-eosin (HE) and Pagoda Red | Brain, heart, coronary arteries, lung, liver, and kidneys | Group D: all cases in this group showed a significant increase in eosinophils, mast cells, and degranulated mast cells within the spleen, with the degranulated mast cells predominantly located in the splenic sinuses. | Postmortem serum from femoral blood | Group A: In only one case, a β-tryptase concentration of 65 ng/mL was recorded. Group B: In only one case, a β-tryptase concentration of 45 ng/mL was recorded. Group C: In three cases, the β-tryptase concentrations were 51 ng/mL, 46 ng/mL, and 48 ng/mL. Group D: β-tryptase concentrations ranged from 146 to 979 ng/mL. | 45 ng/mL | Total IgE, cardiac troponin I, N-terminal pro-brain natriuretic peptide (NT-proBNP), procalcitonin, and C-reactive protein. Group A: Total IgE levels were lower than the clinical reference value (5-50 kU/L) in all cases. Group B: Total IgE levels were lower than the clinical reference value (5-50 kU/L) in all cases. Group C: Total IgE levels were lower than the clinical reference value (5-50 kU/L) in all cases. Group D: Total IgE levels were not specifically reported for this group, but the study emphasizes that elevated postmortem β-tryptase levels, even in the absence of increased total IgE, can indicate fatal anaphylaxis—highlighting that total IgE is not a consistently reliable marker for diagnosing anaphylactic deaths postmortem. | Not mentioned | Not mentioned | Not mentioned | Descriptive statistical analysis of β-tryptase levels and other biomarkers |
| Comment et al. (2014)  Switzerland (14) | 94 subjects | 6 cases: individuals admitted to local hospitals where they underwent diagnostic procedures with contrast media administration and died between 45 and 90 minutes after, due to suspected acute systemic hypersensitivity reactions to contrast media in all cases. 10 cases: hypothermia fatalities. 10 cases: diabetic ketoacidosis deaths. 10 cases: suicides (gunshot wound to the head). 18 cases: heroin overdoses. 10 cases: cardiac arrhythmias and cardiac arrests. 10 cases: individuals with severe coronary artery atherosclerosis, coronary artery calcifications, and myocardial fibrosis at autopsy, without acute coronary thrombosis or myocardial infarction. 10 cases: individuals with severe coronary artery atherosclerosis, acute coronary thrombosis, and acute myocardial infarction at postmortem examination. 10 cases: multiple traumas (cyclists or pedestrians involved in crashes). 10 cases: deaths unrelated to anaphylaxis, severe postmortem changes, and available pericardial fluids. | Postmortem interval not exceeding 48 hours. Availability of postmortem serum from femoral blood, urine, vitreous humor, and pericardial fluid | Autopsies were performed within 24 hours of death, if possible, or 24 hours after the discovery of the body | In the anaphylactic deaths group, pulmonary congestion and edema were noted, along with widespread visceral congestion. Brain swelling was consistently observed. In three cases, mild to severe atherosclerosis of the coronary arteries was identified. | Hematoxylin-eosin (HE) and Pagoda Red | Brain, heart, lung, liver, kidney, and spleen | Pagoda Red staining revealed characteristic histological patterns indicative of splenic and pulmonary eosinophilia, along with the presence of degranulated mast cells. | Postmortem serum, pericardial fluid, urine, and vitreous humor | In the anaphylactic deaths, the range in blood was 146–979 ng/mL. In the pericardial fluid, concentrations were higher than 45 ng/mL. The highest concentration was 119 ng/mL. Vitreous and urine β-tryptase levels were systematically lower than the clinical reference value (11.4 ng/mL). β-tryptase concentrations in pericardial fluid from bodies with causes of death unrelated to allergic reactions and advanced decomposition ranged from 250 ng/ml to 5150 ng/ml. This suggests that significant postmortem changes are linked to substantial β-tryptase leakage into the pericardial space. | 45 ng/mL | Not mentioned | Performed using anti-tryptase antibodies on spleen samples | Confirmed the presence of degranulated mast cells | Not mentioned | Descriptive statistical analysis of β-tryptase levels in different tissues |
| Guo et al. (2015)  China (22) | 35 | 15 autopsy cases of drug-related fatal anaphylaxis and 20 normal autopsy cases | Not mentioned | Autopsies were performed within 72 hours of death | Not mentioned | Not mentioned | Not mentioned | Not mentioned | Postmortem serum and pericardial fluid | Respectively, 43.50 ± 0.48 (μg/L) and 28.64 ± 0.321 (μg/L) | Not mentioned | Carboxypeptidase A in postmortem serum and pericardial fluid, respectively 8.99 ± 3.911 (μg/L) and 4.34 ± 2.411 (μg/L) | Not mentioned | Not mentioned | Performed using mast cell tryptase and carboxypeptidase A antibodies on stomach, jejunum, heart, lung, and larynx. Less carboxypeptidase A was expressed in tissues from anaphylaxis cases than controls. Multiple tryptase-positive particles were detected in the mucosal layer, with fewer in the muscular layer of the stomach and jejunum in anaphylaxis fatalities. In contrast, tryptase expression was lower in tissues from the control group. Additionally, tryptase expression was observed in other tissues, including the bronchial wall and small vessel walls in the lung, the small vessel walls in the submucosa of the larynx, and the peripheral mesenchyme surrounding small vessels in the heart. | Student's t-test, with statistical significance defined as p<0.05 |
| Xiao et al. (2017)  China (23) | 74 subjects | Group A: 20 cases of anaphylaxis Group ACD: 30 cases of acute cardiovascular disease  Group ADA: 10 cases of acute dissecting aneurysm Group C (controls): 14 cases of bacterial pneumonia and 6 cases of methamphetamine poisoning | The criteria for anaphylaxis included the typical clinical symptoms of allergic reactions such as hypotension, rash, and shock, without positive evidence to support any other apparent cause of death, as well as the following nonspecific autopsy findings: pulmonary congestion and edema, visceral congestion, and occasionally laryngeal edema. | The interval between death and autopsy was 12–72 hours. All corpses were stored at 4 °C within 24 hours of death. | Not mentioned | Not mentioned | Not mentioned | Not mentioned | Postmortem femoral blood | Group A: 12.3–309.0 ng/mL.  The tryptase concentration increased in some autopsy cases of ACD.. Elevated tryptase concentrations were also observed in some ADA cases that were suspected to be anaphylaxis cases before autopsy. Thus, tryptase concentrations of Group ADA were compared with those of the control group, and a significant difference was found between the groups. | 43 ng/mL | Not mentioned | Not mentioned | Not mentioned | Not mentioned | Receiver operating characteristic (ROC) curve analysis for the cutoff values of tryptase concentration and sensitivity and specificity. Kruskal–Wallis U test for nonparametric data. Mann–Whitney test used to compare ACD cases to ACS cases. Spearman’s rank correlation test for the correlation between the tryptase concentration and postmortem interval, gender, and age. |
| Tse et al. (2018)  New Zealand (24) | 65 subjects | 9 cases of anaphylaxis 45 controls | Not mentioned | Not mentioned | Not mentioned | Not mentioned | Not mentioned | Not mentioned | Postmortem femoral blood | A mean of 121.6 μg/L for anaphylactic deaths and 18.1 μg/L for controls | 54 μg/L | Not mentioned | Not mentioned | Not mentioned | Not mentioned | Wilcoxon rank-sum test to compare tryptase levels between groups. Kruskal-Wallis test to analyze nonparametric data (2-tailed p-value <0.05 was considered statistically significant). Univariate logistic regression models to determine predictive values of variables analyzed (tryptase, age, gender, CPR, and PMI) for anaphylactic death. ROC curve analysis to define diagnostic cutoffs (p-value <0.1). |
| Tejedor-Alonso et al. (2020)  Spain (18) | 122 subjects | 122 cases of suspected anaphylaxis death were initially considered, and 46 were selected as fatal anaphylaxis (39 of which had tryptase results available). | Not mentioned | Not mentioned | Not mentioned | Not mentioned | Not mentioned | Not mentioned | Postmortem blood serum (collected within 24 hours after death) | The optimal cutoff for postmortem tryptase in anaphylaxis was 64 µg/L. In cases of drug-induced anaphylaxis and Hymenoptera stings, the cutoff of around 40–60 µg/L is more reliable, while in food-induced anaphylaxis the tryptase values ​​can be even lower. | Not mentioned | Not mentioned | Not mentioned | Not mentioned | Not mentioned | Calculation of ROC (receiver pperating characteristic) curves and use of the Youden index to identify the optimal postmortem tryptase cutoff. Comparisons with nonparametric tests (Kruskal–Wallis, Wilcoxon rank-sum test). Use of logistic regression models to estimate the probability that the death was attributable to anaphylaxis. |
| Feng et al. (2021)  China (15) | 60 subjects | 30 cases of anaphylaxis and 30 cases of other deaths | The causes of death in the selected cases were determined through a systematic and comprehensive approach, including forensic anatomical analysis, histopathological evaluation, toxicological testing, and biochemical examinations. | Elapsed time after death < 48 hours or bodies frozen within 6 hours of death | Not mentioned | Not mentioned | Not mentioned | Not mentioned | Not mentioned | Not mentioned | Not mentioned | Not mentioned | Performed using anti-FcεRIα monoclonal antibody, anti-mast cell tryptase, monoclonal antibody, anti-FcεRIα polyclonal antibody, and anti-mouse IgG antibody on lung samples | A large number of tryptase-positive mast cells were identified in the lung tissues of the drug-induced anaphylactic death group, primarily localized around the bronchioles, bronchi, and blood vessels. In contrast, only occasional tryptase-positive cells were observed in the lung tissues of the control group. Numerous FcεRIα-positive cells were observed in the lung tissue of the drug-induced anaphylactic death group, characterized by abundant brown staining on the cell membranes. These positively stained cells were predominantly located near the bronchioles and small blood vessels. In contrast, only occasional FcεRIα-positive cells were found in the lung tissue of the control group. | Double immunofluorescence staining for FcεRIα and the mast cell marker tryptase was performed on paraffin lung specimens. Double immunofluorescence showed that FcεRIα was coexpressed with tryptase, confirming that these were mast cells. | Means (± standard deviation) of the number of cells positive for each marker (FcεRIα and tryptase) in selected microscopic fields were calculated. Statistical comparison (p < 0.05) to test for differences between anaphylaxis group and controls (statistical tests not specified in detail, probably ANOVA). |
| Wang et al. (2020)  China (16) | 72 subjects | 20 cases of drug-related anaphylactic sudden death and 52 cases of nonallergic death | Not mentioned | The postmortem interval ranged from 0 to 85 days. | In the allergic group, autopsy findings revealed pronounced laryngeal edema, with some cases showing a narrowed glottic fissure. Microscopic indicators of anaphylaxis included marked congestion across multiple organs and nonspecific pathological changes. The mucous glands of the throat exhibited increased activity, while tissue congestion and intestinal muscle spasms were also noted. In the lungs, alveolar walls were thickened, and the alveolar spaces were filled with pink, edematous fluid. | Hematoxylin and eosin (HE) | Not mentioned | Not mentioned | Not mentioned | Not mentioned | Not mentioned | Not mentioned | Performed using anti-human mast cell tryptase and anti-human IgE on throat, lung, and intestine samples | Mast cell–positive cells were primarily located in the laryngeal lamina propria, concentrated around small blood vessels and mucous glands. In the lungs, they were predominantly found surrounding blood vessels, with a few scattered among pulmonary epithelial cells. In the intestine, mast cells were mainly distributed among the mucosal glands and within the connective tissue of the submucosa. Immunoglobulin E–positive colocalized with mast cells. | Immunofluorescent staining and double immunohistochemistry (IHC) demonstrated IgE positivity in the cytoplasm and membrane of mast cells, with scattered granules evident. | The data were analyzed using IBM SPSS 21.0. Unpaired 2-sided Student t-tests, Kruskal–Wallis one-way analysis of variance, and nonparametric Kruskal–Wallis tests were used to compare several means. Spearman’s rank correlation test was used to analyze the correlation between marker contents and the PMI. P < 0.05 was considered statistically significant. |
| Bonetti et al. (2015)  Italy (25) | 55 subjects | 15 cases of anaphylaxis and 40 cases of known, nonallergic causes of death | Not mentioned | Not mentioned | Autopsy showed pulmonary oedema and generalized visceral congestion. Splenomegaly and brain swelling were also frequently observed. | Hematoxylin-eosin (HE) and Pagoda Red | Spleen | Splenic eosinophils, mast cells, and degranulated mast cells were observed, with degranulated mast cells mainly located in splenic sinuses. | Postmortem femoral blood | β-tryptase levels were higher than the clinical reference value | 13.5 μg/L | Total IgE and specific IgE levels exceeded the clinical reference value (5–50 kU/L). | Performed using anti-tryptase antibodies on spleen samples | Confirmed the presence of mast cells in the spleen | Not mentioned | Mann–Whitney U test (group comparison). Spearman’s rankcorrelation (age, gender, biochemical and cellular markers). Significance threshold p<0.05. |
| Esposito et al. (2021)  Italy (26) | 22 subjects | 11 cases of anaphylatic death and 11 controls | Cases with weak or missing information about the manner of death were excluded. Decomposed bodies were excluded from the study. | Autopsies were performed within 4 days of death | Pulmonary swelling and edema were observed in all cases during autopsy. Macroscopic examination revealed that 64% of the cases showed pharyngeal or laryngeal edema along with mucus obstruction in the airways. A skin reaction was noted during external examination in only one case (9%). | Hematoxylin-eosin (HE) | Lungs, glottis, and skin | All cases displayed pulmonary congestion and edema | Postmortem femoral blood | Ranged from 40.5 μg/L to 640 μg/L | Not mentioned | Not mentioned | Performed using anti-tryptase antibodies on lungs, skin, and glottis | All cases showed strong immunopositivity for anti-tryptase antibody staining. Samples taken from the lungs, the skin at the site of medication injection, and the glottis demonstrated strong and widespread immunolabeling with anti-tryptase antibodies. In particular, lung tissue revealed the presence of anti-tryptase in mast cells located within the connective interstitium and bronchiolar structures. The skin at the injection site also exhibited intense mast cell antibody staining within the connective dermis. Additionally, the glottis showed marked overexpression of anti-tryptase staining, distributed throughout the laminar connective tissue at the level of the vocal folds. | Not mentioned | Comparison of serum tryptase levels in cases versus reference ranges. Densitometric comparison (pixel 2) to quantify immunohistochemical positivity in anaphylaxis cases versus controls. Use of statistical tests (Shapiro–Wilk test for normality, nonparametric t-tests), and presentation of results as means ± SD. |

TABLE S4 Synthesis table of the studies analyzed.

| **Authors and country** | **Age, sex, and anamnesis** | **Suspected condition** | **Suspected triggering agent** | **Immediate death** | **Autopsy, putrefaction, and  sample collection time.** | **Autopsy findings** | **Tryptase research** | **Tryptase values** | **Reference cutoff** | **Technique used** | **Histological findings** | **Results of fluid investigations** | **Other fluid investigations** | **Immunohistochemistry** | **Results of immunohistochemistry on tissue** |
| --- | --- | --- | --- | --- | --- | --- | --- | --- | --- | --- | --- | --- | --- | --- | --- |
| Tambuzzi et al. (2021)  Italy (27) | A 30-year-old man was discovered deceased in his prison cell next to partially eaten peaches with exocarp. He had a history of drug addiction, heavy smoking, asthma attacks, and an unspecified pollen allergy. No food allergies were reported. | Anaphylaxis | Peaches | Not mentioned | Autopsy was performed three days after death | The external examination showed no evidence of traumatic injuries. A farewell note was discovered in the deceased’s pocket. Upon examining the oral cavity, mild redness of the oral and labial mucosa was noted. The autopsy revealed pulmonary, hepatic, and bilateral renal congestion, along with liquid blood and reddish mucus in the trachea and bronchi. There were no signs of glottic edema, urticaria, or other skin reactions typically associated with allergic responses. No food particles were found in the airways, and no pathological findings were noted in other organs. | Serological examination | 15.7 μg/L | The reference value in healthy individuals is between 5.6 and 9.8 μg/L, according to the literature | Hematoxylin–eosin staining | The glottis, particularly on the right side, showed moderate chronic inflammatory cellular infiltrates, consisting of lymphocytes, plasma cells, and cells suggestive of mast cells. Vascular dilatation, congestion, and mild edematous infiltration of the glottal subepithelial connective tissue were also observed. The lungs, as well as the tracheal and bronchial mucosa, exhibited cellular elements indicative of mast cells. Similar findings were noted in the anterior and posterior walls of the left ventricle and the interventricular septum. Microscopic examination of the gastric contents revealed cellular residues with a vegetal structure and cavitated cellulose scaffolding, which were identified as peach residues due to their morphology and appearance. | Postmortem IgE tests revealed a high titer of IgE antibodies (154 kU/L) in the patient's femoral blood. Additional tests showed moderately elevated to very high levels of specific IgE against peach and birch tree antigens. | Toxicological investigations were negative for both the blood alcohol content and substances of abuse. A significant presence of salicylic acid (130 ng/L), which is notably found in peaches, was detected in the gastric contents. | Samples of the glottis, lungs, and myocardium  Anti-CD117 antibodies and especially anti-tryptase antibodies were used | A significant number of cells with cytoplasm rich in granules reactive to both applied antibodies were identified in the glottis. This finding was consistent with the presence of intact (non-degranulated) mast cells located in the subepithelial connective tissue between the glands and within the connective tissue separating the striated muscle fibers of the true vocal cords. Additionally, several cells with rounded nuclei and abundant cytoplasm but lacking granules were observed, which were interpreted as degranulated mast cells. Around these cells, numerous anti-Around these cells, numerous anti-tryptase-positive granules were dispersed throughout the subepithelial connective tissue and among the muscle fibers, forming a distribution pattern described as “yard-like”. Similar findings were noted in samples from the lungs and myocardium. In both tissues, positive staining for anti-tryptase and anti-CD117 was observed; this wasmore pronounced in the lung samples than in those from the myocardium. |
| Mondello et al. (2023)  Italy (31) | A 59-year-old Caucasian man with a history of sensitization to Hymenoptera stings, which previously resulted in facial edema following both bee and wasp stings. Sensitization was later confirmed through skin testing. His medical history was otherwise unremarkable, with no evidence of cardiovascular or respiratory diseases. | Anaphylaxis | Arthropod sting (Hymenoptera) | Not mentioned | Autopsy was performed 24 hours after death | External examination showed no evidence of bee stings. Gross anatomical inspection revealed mild laryngeal edema, the presence of a whitish foamy fluid in the bronchial tree, and a dense, red-brownish, foamy fluid within the lungs. | Serological examination of peripheral blood | 189 μg/L | Not mentioned | Not mentioned | Routine histological examination revealed subacute pulmonary emphysema, intra-alveolar edema and hemorrhage, pronounced congestion of the interalveolar septa, bronchospasm, and areas of bronchial obstruction due to excessive mucus production. Myocardial tissue exhibited hypertrophic myocytes, myofiber fragmentation, and focal wavy fibers. Atherosclerotic plaques were also present in the coronary arteries. | The ImmunoCAP method was used to determine total IgE levels, which measured 200 kU/L. Specific IgE testing was also performed for honey bee, white-faced hornet, common wasp, paper wasp, and yellow hornet allergens. Results showed elevated IgE levels for honey bee (5.30 kUA/L) and yellow jacket (3.00 kUA/L). | Toxicological analysis was negative for alcohol, illicit substances, and psychotropic drugs. | Samples of larynx, lung, heart, and spleen tissue  Primary anti-human antisera monoclonal mouse anti-tryptase antibody and biotinylated goat anti-mouse IgG secondary antibody | Immunohistochemical analysis revealed intense expression in the larynx and lungs, with numerous immunopositive mast cells and widespread positivity for degranulated tryptase. Mild expression was observed in the walls of the coronary arteries and myocardial tissue, characterized by scattered mast cells and focal tryptase degranulation. Similarly, mild positivity was noted in splenic tissue, with the presence of mast cells and diffuse expression of degranulated tryptase. |
| McKibbin et al. (2021)  Canada (34) | A male teenager died due to peanut exposure from oral intercourse. The deceased had a history of IgE-mediated peanut allergy, allergic rhinitis, and severe asthma. | Anaphylaxis | Peanuts | A day after exposure | No mention is made of whether or not an autopsy was performed | Not mentioned | Serological examination on peripheral blood sampled antemortem | 47.0 μg/L | Normal reference range 3.8–11.4 μg/L | Not mentioned | Not mentioned | Not mentioned | Toxicological analysis did not find any evidence of drugs of abuse. | Not mentioned | Not mentioned |
| Panata et al. (2020)  Italy (32) | A 57-year-old man was reportedly stung by an insect while gardening. He had never suffered from an allergy or anaphylaxis. | Anaphylaxis | Arthropod sting (Hymenoptera) | A few hours later | Autopsy was performed 42 hours after death  No putrefaction | External examination revealed a small wheal measuring 5 mm in diameter with surrounding erythema of approximately 10 mm on the right parietal region of the scalp. Autopsy findings included edema of the brain, pharynx, larynx, and trachea, accompanied by reddish mucus and whitish foam. Upon opening the thoracic cavity, the lungs were found to completely cover the pericardium. They appeared regular in shape and consistency, with increased volume and a reddish coloration. The large and medium bronchi contained abundant whitish foam. | Serological examination of peripheral blood | 142 μg/L | 45 μg/L, as proposed by some authors | Not mentioned | Histological examination revealed severe alveolar edema interspersed with areas of acute pulmonary emphysema. Additionally, edema was observed in the epiglottis, vocal cords, and the submucosal layer of the trachea, accompanied by marked vascular congestion. | Not mentioned | 1.61 g/L of alcohol | Lung and skin samples  Antibodies anti-tryptase and anti-mast cells (CD117, c-kit) | A significant number of degranulating mast cells, with tryptase-positive material present outside the cells, were observed in the lungs. Skin samples from the right parietal region of the head showed an increase in interstitial and perivascular mast cells, with tryptase-positive material located outside the cells. |
| D'Errico et al. (2020)  Italy (42) | A 16-year-old boy with a medical history of allergic asthma, celiac disease, and known food-induced allergies to fish, fresh milk, peanuts, hazelnuts, walnuts, apples, kiwis, and peaches. | Anaphylaxis | Food | Not mentioned | Autopsy was performed 2 days after death  No putrefaction | External examination was unremarkable. Autopsy revealed subpleural petechiae and heavy lungs with white foam present in the main bronchi. Mild cerebral edema was also noted. The heart appeared normal in conical shape with minimal subepicardial fat. The coronary arteries were patent, with no evidence of luminal obstruction. No macroscopic pathological findings were observed in the laryngo-tracheo-bronchial tree. The stomach contained approximately 200 cc of partially digested food. | Serological examination of peripheral blood | 41.4 mg/L | Normal value ⩽10 mg/L | Hematoxylin–eosin staining | Histological examination revealed widespread acute vascular stasis, mild cerebral edema, and interstitial myocardial edema. Additionally, acute pulmonary edema was noted, interspersed with areas of acute pulmonary emphysema. Intraparenchymal hemorrhages were observed in both the spleen and adrenal glands. | Postmortem specific IgE testing confirmed the anamnestic history of sensitization to multiple food allergens. Notable findings included elevated IgE levels for cod parvalbumin (34.5 kUA/L), tropomyosin (40.5 kUA/L), Brazil nut (5.84 kUA/L), omega-5-gliadin from wheat-derived foods (1.07 kUA/L), and gluten (54.8 kUA/L). | Negative toxicological analysis results | Lung tissue  Anti-tryptase antibody | Pulmonary mast cells were identified and quantified, revealing a high number of degranulating mast cells with extracellular tryptase-positive material. Quantitative analysis demonstrated a significant increase in pulmonary mast cell density in this case, with an average count of 12,551 cells per 100 mm², compared to the control group of traumatic deaths, which showed an average of 3,557 cells per 100 mm². |
| Tse et al. (2018)  New Zealand (40) | A 66-year-old man died after antibiotic administration. He was known to be allergic to Tazocin and also had a significant background medical history including ischemic heart disease, valvular heart disease, and chronic obstructive airways disease. | Anaphylaxis | Antibiotics | Within 1 hour of antibiotic administration | Autopsy was performed 6 days after death  No putrefaction | External examination showed previous cardiac surgery but was otherwise unremarkable. The upper airway was markedly edematous, especially around the epiglottis and the trachea. The heart was enlarged with an uncomplicated aortic and mitral valve repair and an old myocardial infarction in the posterior free wall of the left ventricle. The lungs showed chronic obstructive pulmonary disease with emphysematous changes. No pneumonia was identified. | Serological examination of peripheral blood | The admission/baseline tryptase was 5.6 μg/L. The 2 consecutive postmortem tryptase levels were grossly elevated at 522 μg/L on day 3 after death but decreased to 300 μg/L on day 6 after death | Not mentioned | Not mentioned | Edema with mast cell infiltration | IgE levels were 783 kU/L, specific IgE for Penicilloyl G was 0.78 kU/L, and specific IgE for Penicilloyl V was 1.15 kU/L. | Not mentioned | Not mentioned | Not mentioned |
| Ichimata et al. (2020)  Japan (35) | A 50-year-old man suddenly died after taking loxoprofen sodium 1 day before his death for a right shoulder pain. He had complained of mild digestive symptoms after taking other NSAIDs months before. He had no risk factors for cardiovascular disease except for cigarette smoking, no history of allergic disorders, and no symptoms suggestive of cardiac or respiratory disorders. | Eosinophilic coronary periarteritis (ECPA) | Loxoprofen sodium | Not mentioned | Not mentioned | The right and left lungs exhibited severe edema and congestion. The spleen also showed severe congestion. No gross abnormalities were observed in other organs. Histopathological examination revealed findings consistent with bronchial asthma, including hypersecretion of the bronchial epithelium, mucus plug formation, thickening of the basement membrane, and eosinophilic infiltration, observed in localized areas of both lungs. Severe congestion was confirmed in both lungs and the spleen. Additionally, mild edema and eosinophilic infiltration were noted in the intestinal tissue. | Serological examination of peripheral blood | 47.8 μg/L | Normal <11.4 μg/L | Not mentioned | Histological examination revealed eosinophil-predominant inflammation in the three main branches of the coronary arteries, without evidence of fibrinoid necrosis or granulomatous changes in the adventitia or surrounding soft tissues. Moderate medial and adventitial fibrosis was observed, along with fibrous thickening of the intimal layer. The degree of luminal narrowing was approximately 70% in the right coronary artery and around 40% in both the left anterior descending and left circumflex arteries. Inflammation was more pronounced in the right coronary artery compared to the other branches. Small numbers of eosinophils were also noted within the intimal layer. Additionally, focal fragmentation of the external elastic lamina, accompanied by fine adventitial and medial fibrosis, was identified. | Histamine: 134 μg/L  Reference range 0.11 - 0.50 μg/L | Toxicology testing was negative for ethanol and major drugs. Loxoprofen sodium was detected in the blood (0.76 mg/L). | Human mast cell tryptase | Many positive mast cells were evident in the perivascular tissue containing peripheral nerve trunks. |
| Ihama et al. (2014)  Japan (39) | A 40-year-old female died after injuring her finger on the spine of a starfish. She had no medical history of other allergic diseases, such as bronchial asthma, and had previously been stung 5 times by the same species. | Anaphylaxis | Crown-of-thorns starfish | 13 hours after exposure | Autopsy was performed 28 hours after death  No putrefaction | She had been suffering from significant swelling (edema) of the skin and the conjunctiva around her face, to the point that the bulbar conjunctiva showed a slight yellowish discoloration (jaundice). A sting from a crown-of-thorns starfish on the top of her right middle finger led to severe swelling and discoloration of the finger, swelling of the upper right arm, and visible hemoglobin leakage along the blood vessels of her forearm and hand. Examination of her respiratory system showed marked swelling of the laryngopharynx, which had narrowed her airway. Her lungs were severely swollen and congested, weighing 405 and 420 g respectively, and there was a substantial buildup of fluid in both the pleural cavity and abdominal cavity (ascites). No congenital heart abnormalities were found, and there was no evidence of coronary artery narrowing (stenosis) or ischemic damage to the heart muscle (myocardium). On gross examination, the liver appeared soft and swollen, with poorly defined borders between the hepatic lobules. | Serological examination of peripheral blood | 6.7 μg/mL | Normal <13.5 μg/mL | Not mentioned | Microscopic examination of the liver showed widespread hepatocellular necrosis across the parenchyma and lobules, along with sinusoidal dilation due to congestion, the presence of bile plugs in the canaliculi, and eosinophilic cytoplasm with pyknotic nuclei. In the lungs, microscopic analysis revealed alveolar hemorrhage and eosinophilic infiltration, as well as significant aggregation of neutrophils and eosinophils within the blood vessels. Notably, there was a marked concentration of eosinophils around the bronchi and extensive infiltration of the alveoli. | Histamine: 1030 ng/mL (normal 0.15–1.23 ng/mL). Non-specific IgE: 95.6. IU/mL (normal <170 IU/mL). Laboratory tests at the time of admission revealed severe hemolysis, hemoconcentration, and abnormal hemostasis. Extremely elevated levels of liver enzymes, including aspartate transaminase, alanine aminotransferase, and lactate dehydrogenase, indicated significant liver damage shortly after injury. | Toxicology testing was negative for ethanol and major drugs. | Not mentioned | Not mentioned |
| Radheshi et al. (2016)  Italy (33) | A 55-year-old woman died after taking antibiotics. In the days leading up to her death, she reported symptoms of upper respiratory discomfort, including fever and a productive cough. Given her history of bacterial bronchitis a few months earlier, the physician prescribed the same antibiotic that had been used during her previous illness. | Anaphylaxis | Antibiotics | Within 2 hours of antibiotic administration | Autopsy was performed 96 hours after death. The corpse remained at room temperature for 36 hours. | External examination showed signs of decomposition, including widespread green discoloration of the skin and marbling of both the upper and lower extremities. Despite decomposition of the internal organs, mild laryngeal edema remained observable. The heart showed no signs of hypertrophy or dilation, and the myocardium was free of fibrosis or ischemic lesions. The coronary arteries followed a normal anatomical course and demonstrated mild atherosclerotic changes without significant narrowing. Similarly, the aorta exhibited mild atherosclerosis. The lungs were notably edematous and congested, the spleen was enlarged and congested, and the brain showed signs of edema. No other abnormalities were identified. | Serological examination of peripheral, aortic, and right heart blood | 46 μg/L, 49 μg/L, and 42 μ/L | Not mentioned | Hematoxylin–eosin and Pagoda Red (spleen) staining | Tissue samples from the heart, lungs, kidneys, liver, brain, and spleen were processed routinely. This revealed decompositional changes in most organs. Splenic eosinophils and mast cells, as well as degranulated mast cells, were detected (Pagoda Red), mainly located in spleen sinuses. | Specific IgE anti-clarithromycin could not be measured due to postmortem serum unavailability. | Toxicology testing was negative for ethanol and major drugs. | Spleen samples  Anti-tryptase antibodies | The presence of mast cells in the spleen was confirmed. |
| Sravan et al. (2015)  Australia (41) | A 62-year-old man was bitten by an ant. He had a complex medical history of atopic disease, including early childhood asthma, previous anaphylactic reaction to ant bites, and indolent mastocytosis (baseline antemortem serum tryptase level of 40 ug/L). He also had a medical history of mitral valve replacement, type II diabetes mellitus, peripheral vascular disease, alcoholism, and treated head and neck squamous cell carcinoma. | Anaphylaxis with a background of indolent mastocytosis | Ant bite | Not mentioned | Autopsy was performed 3 days after death  No putrefaction | There were no visible ant bite marks on the skin, and no gross cutaneous signs indicative of anaphylaxis or mastocytosis were observed. | Serological examination of two different peripheral blood samples, drawn 2 and 3 days after death | 130.0 μg/L and 84.4 μg/L | Not mentioned | Not mentioned | Histological analysis of the abdominal skin revealed an increased number of interstitial and perivascular mast cells in the dermis, which tested positive for c-kit. In contrast, histological examination of the bone marrow was normal, with no evidence of mastocytosis. Examination of the upper airways revealed swelling and edema of the soft palate, uvula, and both the true and false vocal cords, accompanied by mucosal inflammation and submucosal edema. Microscopic analysis showed prominent infiltration of mast cells, which were c-kit positive. | Immunological assays for IgE-specific allergens for common food and insect venoms were inconclusive. | Not mentioned | Not mentioned | Not mentioned |
| Gilbert et al. (2017)  Australia (60) | A 33-year-old woman died after injecting herself with a cannabis solution. She had been a regular user of amphetamines and cannabis. There was a history of epilepsy and a diagnosis of Huntington’s disease. | Anaphylaxis | Intravenous injection of aqueous cannabis extract | Not mentioned | Not mentioned | There was no evidence of facial swelling or edema of the upper airway mucosa. Neuropathological examination of the brain revealed focal areas of glial scarring in the right prefrontal cortex and left uncus, consistent with a prior head injury. Additional changes observed in the temporal cortex and cerebellum aligned with the clinical history of epilepsy. Ubiquitinated intranuclear inclusions were identified in neurons of the cerebral cortex and basal ganglia, consistent with Huntington’s disease. | Serological examination of peripheral blood | 200 μg/L | <12 μg/L | Not mentioned | Histological examination revealed eosinophil infiltration in the bronchial submucosa, with increased numbers also observed in the spleen, pulmonary capillaries, and hepatic sinusoids—findings suggestive of peripheral eosinophilia, raising the possibility of an underlying allergic disorder. No evidence of trauma was identified aside from that associated with resuscitation efforts. | Allergen-specific IgE testing for cannabis was not available. However, testing for various fungal organisms was negative. | Toxicological analysis revealed non-lethal concentrations of methylamphetamine and amphetamine, consistent with illicit methylamphetamine use. Additionally, 1 mg/L of THC and 3 mg/L of its metabolite, 11-nor-9-carboxy-THC, were detected. The syringe used by the decedent shortly before her collapse was found to contain Δ-9-THC. | Not mentioned | Not mentioned |
| Kitulwatte et al. (2017)    Sri Lanka (36) | A 52-year-old male died after the administration of an intravenous ceftazidime injection. He was a known diabetic and allergic to amoxicillin and ampicillin. | Kounis syndrome | Antibiotics | 19 hours after ceftazidime administration | Not mentioned | An autopsy examination showed a surgically altered wound over the right sole, massive laryngeal oedema, and mucous plugging with collapsed lungs. The authors also observed an enlarged spleen and blood-stained, partially digested food in the stomach, associated with submucosal erosion. The coronary arteries showed 30%–40% atheroma. | Serological examination of peripheral blood | 118 μg/L | <11.4 μg/L | Not mentioned | The examination revealed myocardial cellular infiltration, predominantly composed of neutrophils, with some mast cells and eosinophils. The myocytes exhibited early signs of contraction band necrosis, and the myocardial changes were transmural in nature. Mucous blockage was observed in the bronchi, accompanied by moderate peribronchial cellular infiltration, including some eosinophils. The liver showed signs of moderate fatty degeneration. No hypoxic changes were noted in the brain. | Not mentioned | Not mentioned | Not mentioned | Not mentioned |
| Wen et al. (2021)  Japan (37) | A male in his 50s with a diagnosis of rectal cancer and multiple metastases in the liver and lymph nodes died after initial cetuximab administration. He had a history of cerebral infarction, hypertension, and hyperlipidemia. | Anaphylaxia | Chemotherapics (cetuximab) | 2 hours after the administration | Autopsies were performed 1 hour and 72 hours after death.  No putrefaction | The first autopsy revealed no pathological condition, such as pulmonary embolism, cerebral infarction, or myocardial infarction, which may have caused sudden respiratory arrest. Autopsy and histopathological examination revealed remote myocardial infarction and advanced coronary atherosclerosis, without evidence of acute inflammation or hemorrhage. Additionally, severe atherosclerosis with ulceration was noted extending from the distal abdominal aorta to the proximal segments of both the left and right common iliac arteries. No significant abnormalities were found in the other organs. | Serological examination of peripheral blood | Before treatment: 1.8 ng/mL  After treatment: 258 ng/mL | 1.0–1.5 ng/mL | Not mentioned | The rectal tumor was histopathologically identified as adenocarcinoma. The hepatic tumor was histopathologically identified as metastatic rectal cancer. A white tumor was found outside the hilum of the left kidney and was histopathologically identified as a Fuhrman grade-2 clear cell renal cell carcinoma. | Before treatment, the bovine thyroglobulin-specific IgE level was 24.9 allergy units (AU)/mL (class 4); beef specific IgE level was 1.28 AU/mL (class 2); and cetuximab-specific IgE was detected. | Low concentrations of the following drugs were detected: amlodipine, carvedilol, chlorpheniramine, chlorpromazine, estazolam, etizolam, lidocaine, phenobarbital, valproic acid, and zolpidem. | Not mentioned | Not mentioned |
| du Toit-Prinsloo et al. (2016)  South Africa (29) | A 38-year-old male was stung by numerous bees. | Anaphylaxis | Arthropode sting | Not mentioned | Not mentioned | External examination revealed approximately 25 sting sites, presenting as small, raised red wheals on the abdomen, along with two sting marks on the right forearm, two on the left upper arm, and three on the right ankle. The epiglottis and surrounding areas showed no significant mucosal redness and only minimal edema. Evidence of medical intervention included a fractured sternum and anterior fractures of the 4th and 5th ribs on both sides, accompanied by some intercostal bruising. Additionally, there was a patchy, film-like subarachnoid hemorrhage over the brain and a bruised subendocardial hemorrhage in the left ventricular outflow tract. An incidental comorbid finding was a well-defined, encapsulated tumor in the left adrenal gland. | Serological examination of peripheral blood sampled 15 hours postmortem | 70.2 μg/L | <10 μg/L | Not mentioned | Histological analysis identified the adrenal tumor as a pheochromocytoma. Lung tissue sections showed small numbers of eosinophils along with some intra-alveolar macrophages. Examination of skin from one of the sting sites revealed mild dermal edema, vascular congestion, and some red blood cell leakage into the underlying fibro-fatty tissue, but no evidence of inflammatory cell infiltration. | Venom IgE antibodies were detected with a level of 3.2 kUA/L (normal level being <0.35 kUA/L). | Not mentioned | Not mentioned | Not mentioned |
|  | A 58-year-old male was stung by a bee. He had a known bee allergy. | Anaphylaxis | Arthropode sting | Not mentioned | Not mentioned | Careful external examination of the body revealed signs of medical intervention; however, no visible bee stings or other abnormalities were detected macroscopically. Internal examination showed no swelling or redness of the laryngeal structures. The heart was markedly enlarged, with concentric left ventricular hypertrophy. There were no apparent atherosclerotic changes in the coronary arteries or other signs of ischemic heart disease. As such, no clear macroscopic cause of death could be determined at autopsy. | Serological examination of peripheral blood sampled 96 hours post mortem | >200 μg/L | <10 μg/L | Not mentioned | Histological examination of tissue slides showed only nonspecific findings of edema and congestion of the lungs and most other parenchymal organs. | Bee venom IgE antibody level was 0.04 kUA/L (normal level being <0.35 kUA/L) | Not mentioned | Not mentioned | Not mentioned |
|  | A 58-year-old male was stung by bees. | Anaphylaxis | Arthropode sting | Not mentioned | Not mentioned | External examination identified five remaining bee stingers embedded in the skin (three on the upper eyelids and two on the cheeks) accompanied by mild periorbital swelling, primarily affecting the upper eyelids. Internal examination once again revealed no noticeable edema or erythema of the glottis or laryngeal structures. No other significant macroscopic abnormalities were observed. | Serological examination of peripheral blood sampled 6 hours postmortem | 49 μg/L | <10 μg/L | Not mentioned | Histological examination likewise revealed only nonspecific systemic and organ changes, with no evidence of inflammatory infiltration at the sting sites or in any other tissues. | Bee venom IgE antibody level was 23.5 kUA/L (normal level being <0.35 kUA/L) | Not mentioned | Not mentioned | Not mentioned |
| Kobek et al. (2014)  Poland (28) | A 52-year-old female died after the administration of intravenous urografin 76%. She had a known history of diabetes, obesity, arterial hypertension, ischemic heart disease, and hypothyroidism after subtotal thyroidectomy. | Anaphylaxis | Urografine 76% | 1.5 hours after administration | Not mentioned | The autopsy findings included obesity and evidence of medical intervention, indicated by injection marks on the neck, left subclavicular area, cubital fossa, and right thigh, along with signs of electrical cardiac defibrillation. Additional findings included subpleural petechiae, mucosal edema of the larynx, most prominently involving the epiglottis and piriform sinuses, hepatic steatosis (fatty liver), and evidence of a previous subtotal thyroidectomy. | Serological examination of peripheral blood and vitreous body | Respectively, >200 μg/L and <1.00 μg/L | <20 μg/L | Not mentioned | Histopathological examination revealed marked submucosal edema in the larynx—particularly in the epiglottis and piriform sinuses—characterized by homogeneous eosinophilic, PAS-positive material and scattered small infiltrates of polymorphonuclear cells. The spleen showed abundant eosinophils within the red pulp. Lung tissue exhibited acute hyperinflation, focal edema, and hemorrhage into the alveolar spaces. The liver showed diffuse fatty changes consistent with hepatic steatosis. | IgE at a concentration of 3.26 IU/ml (normal range < 5.04 IU/ml) | Toxicology testing was negative for ethanol and major drugs. | Not mentioned | Not mentioned |
|  | A 22-year-old male died after eating a candy bar containing peanuts. | Anaphylaxis | Peanuts | About 1.5 hours after administration | Not mentioned | External inspection and postmortem examination revealed cerebral congestion and edema with evidence of tonsillar herniation, along with laryngeal edema, pulmonary edema, and acute lung hyperinflation. Findings also included signs of medical intervention consistent with resuscitation efforts. | Serological examination of peripheral blood | >200 μg/L | <20 μg/L | Not mentioned | Histopathological examination revealed several significant abnormalities. In the brain, there was marked congestion and edema. The lungs exhibited congestion, edema, and areas of acute hyperinflation. The main bronchus showed mucosal congestion and edema, with infiltrates of polymorphonuclear cells, as well as relatively regularly scattered individual eosinophils and small clusters of eosinophils within the lumina of numerous congested blood vessels. Additionally, abundant eosinophils were identified within the blood vessels of the pancreas and in the colonic mucosa. | IgE at a concentration of 1260 IU/ml (normal range < 100 IU/ml) | Toxicology testing was negative for ethanol and major drugs. | Not mentioned | Not mentioned |
| Burkhardt et al. (2019)  Switzerland (38) | A 64-year-old-man died after eating a suspected seafood pie. He had a known specific food allergy to seafood and a diagnosis of epilepsy, as well as learning difficulties since childhood, as a result of meningitis. | Anaphylaxis | Seafood (suspected) | Not mentioned | Autopsy was performed 4 days after death | The macroscopic examination revealed swelling of the larynx with hemorrhagic infiltrates, as well as gastric contents in the esophagus and upper respiratory tract. Additionally, signs of resuscitation were evident, including costal and sternal fractures and injection marks on the upper limbs. | Serological examination of peripheral blood sampled antemortem | 21.9 μg/L | <13.5 μg/L | Pagoda Red staining | The spleen showed scattered eosinophilic reaction and diffused activated mast cells. | Analysis of the antemortem serum and pericardial fluid collected during the autopsy revealed elevated levels of an IgE antibody (46.4 kU/L) reactive to a fish/shellfish mix, while the antibody specific to fish was found to be at a nonsignificant level (< 0.35 kU/L). | Toxicology testing was negative for ethanol and major drugs. | Samples from lungs  Anti-tryptase and anti-CD117 | Immunohistochemical analysis showed a strong positivity for anti-tryptase and anti-CD117 antibodies, indicating a significant presence of activated mast cells. |
| Mercurio et al. (2018)    Italy (30) | A 17-year-old died after eating dinner. He had a known allergy to cow's milk protein and had experienced multiple episodes of bronchospasm and cyanosis, triggered by the ingestion of milk and dairy products. | Anaphylaxis | Not mentioned | About 1.5 hours after eating | Autopsy was performed 13 hours after death | The examination revealed normal cadaveric rigidity in all muscle groups, with hypostasis present on the face, upper chest, and limbs, slightly fading under digital pressure. Petechiae were observed in the eyes, and blood was found coming from the mouth and the right nostril. The autopsy revealed edema in the mucosa of the larynx and trachea, accompanied by reddish mucus and white foam. Upon opening the chest, the lungs were found to completely cover the pericardium, and the pleural spaces were devoid of fluid. The right lung had a regular shape and consistency, with increased volume and a reddish hue. The left lung also showed a regular shape, increased volume, and a reddish color. The large and medium bronchi were intact and contained abundant white foam, while the arterial and venous vessels had intact walls. | Serological examination of peripheral blood sampled 5 hours postmortem | 120ng/L | <11.4 ng/L | Not mentioned | Histopathological examination showed the presence of inflammatory cells at the submucosal site of the glottis and in the spleen; there was also evidence of pulmonary acute emphysema. | Examination aimed at detecting IgE confirmed the existence of milk allergy and its derivatives (allergen-specific IgE concentrations above 0.1 kUA/L indicate the presence of sensitization): total IgE 403 kU/L, nBosd4 maltotalbumin milk 4.39 kUA/L, nBosd betalattoglobulin milk 4.84 kUA/L, nBosd 8 casein 95.1 kUA/L, and milk 79.2 kUA/L. | Not mentioned | Not mentioned | Not mentioned |
